# A cost-effectiveness analysis of respiratory syncytial virus (RSV) prophylaxis in infants in the United Kingdom

**DOI:** 10.1186/2191-1991-3-18

**Published:** 2013-08-06

**Authors:** Anthony Bentley, Ivana Filipovic, Katherine Gooch, Katharina Büsch

**Affiliations:** 1 Health Economics, Abacus International, 6 Talisman Business Centre, Talisman Road, Bicester, Oxfordshire OX26 6HR, UK; 2 Health Economics and Outcomes Research, AbbVie, Vanwall Road, Maidenhead SL6 4XE, UK; 3 Global Health Economics and Outcomes Research, AbbVie, 200 Abbott Park Drive Abbott Park, Illinois 60064, USA; 4 Health Economics and Outcomes Research, AbbVie AB, Hemvärnsgatan 9, P.O. Box 1523171 29 Solna, Sweden

**Keywords:** Palivizumab, Prophylaxis, Cost-effectiveness, Respiratory syncytial virus, United Kingdom

## Abstract

**Background:**

Respiratory syncytial virus (RSV) is a common cause of respiratory infection that is highly prevalent in infants. Severe cases of RSV infection require hospitalisation; this is most likely to occur in infant populations at high risk. The study assesses the cost-effectiveness of palivizumab versus no prophylaxis in infants at high risk of hospitalisation with RSV in the United Kingdom (UK).

**Methods:**

A decision tree model was developed to reflect the clinical pathway of infants at high risk of severe RSV infection who receive either prophylaxis with palivizumab or no prophylaxis. The main outcome was the incremental cost-effectiveness ratio (ICER). One-way and probabilistic sensitivity analyses were performed to assess the degree of uncertainty surrounding the results. A threshold analysis considered the impact of clinical and environmental risk factors on the cost-effectiveness in the subgroup of preterm infants 33–35 weeks gestational age (wGA).

**Results:**

Prophylaxis with palivizumab compared with no prophylaxis is associated with the following ICERs; £33,216 for infants with congenital heart disease; £19,168 for infants with chronic lung disease; £3,845 for preterm infants < 29 wGA; £30,205 for preterm infants 29–32 wGA; and £99,056 for preterm infants 33–35 wGA. One-way sensitivity analysis suggests that these results are highly sensitive to the input data. Threshold analysis in the preterm 33–35 wGA subgroup demonstrates that an adjusted RSV-hospitalisation baseline risk of 17.94% or higher would result in an ICER below the £30,000 per quality-adjusted life-year threshold.

**Discussion:**

Palivizumab is cost-effective compared to no prophylaxis in the United Kingdom in many of the subgroups considered, showing that palivizumab would be a cost-effective use of National Health Service resources.

## Background

Human respiratory syncytial virus (RSV) is a common virus that causes respiratory tract infections. In the United Kingdom (UK) these infections usually occur between the months of October to March and are characterised by a relatively short epidemic of about six weeks [1]. In the majority of cases, RSV infection is a mild and self-limiting illness, however it can be severe enough to cause lower respiratory tract infection (LRTI) requiring hospitalisation in babies and infants and is associated with significant respiratory morbidity including bronchiolitis, pneumonia and even death [1,2]. 2-3% of infants aged < 1 year are admitted to hospital annually with RSV bronchiolitis [3]. Muller-Pebody et al. suggested that 17.5% of hospital admissions for LRTIs and 74.8% of admissions for unspecified bronchiolitis were caused by RSV. They further suggested that 28.3/1000 hospital admissions under one year of age were attributable to RSV [4]. Infants with underlying medical conditions such as chronic lung disease (CLD), congenital heart disease (CHD) or who were born prematurely are particularly at increased risk of complications from RSV infection, resulting in prolonged hospitalisation, admission to intensive care and poorer outcomes [5-7]. A 2003 UK cohort study found that of 304 infants who were either < 36 weeks of gestation and < 6 months of age at the onset of RSV season or < 2 years of age with CLD needing home oxygen therapy, 9.2% were re-hospitalised for RSV disease [3]. Furthermore, there is evidence to suggest that severe LRTI in early childhood may be associated with respiratory morbidity such as recurrent wheeze and/or asthma in later childhood and early adulthood, however mechanisms for this are poorly understood [8,9]. It is well recognised that severe RSV infection is associated with a significant health and economic burden [10-15].

RSV is an RNA virus that is spread via respiratory droplets from nasal secretions of infected individuals, and the risk of infection increases in settings where the chances of exposure are greater [14]. As the virus-contaminated droplets can persist for several hours on surfaces, the risk is particularly high in environments, such as playgrounds, overcrowded housing, and hospitals [14]. Evidence suggests that the majority of infants will have been infected with RSV by the time they reach two years of age [13]. Recurrent infection is frequent, however it is thought that primary infections may provide some protection against future severe disease [13,14]. Mild cases of RSV are usually not formally diagnosed and treatment involves self-care measures to relieve the symptoms [13,14]. Severe cases may lead to hospitalisation and in some cases admission to intensive care. To date there is no effective treatment for RSV LRTI beyond supportive care [13,14].

Palivizumab is a humanised monoclonal antibody approved in Europe, and the only pharmacological prophylaxis licensed in Europe, for the prevention of serious LRTI requiring hospitalisation caused by RSV in children at high-risk of RSV disease [7]. This includes pre-term infants (≤ 35 weeks gestational age [wGA] and aged under six months at the start of the RSV season), infants under two years of age with CLD requiring treatment for bronchopulmonary dysplasia within the previous six months, and infants under two years of age born with haemodynamically significant CHD [13-15].

It is well established that palivizumab prophylaxis is an effective and well tolerated approach to reducing the incidence of LRTI requiring hospitalisation [5,6]. In clinical trials, palivizumab has been shown to reduce the overall incidence of RSV-associated hospitalisations in high-risk pre-term infants and those with CLD and CHD compared with placebo [16,17]. However, it is necessary to evaluate the cost-effectiveness of healthcare interventions. Although studies have previously described the cost-effectiveness of palivizumab in high-risk infant groups [12-15], the conclusions have varied and concerns have been raised regarding the data and assumptions used within these analyses [18].

This study re-evaluated cost-effectiveness of palivizumab in the UK using updated data derived from clinical trials and recent healthcare costs. The most influential parameters and their impact on the incremental cost-effectiveness ratio (ICER) have been investigated to provide a transparent assessment.

## Methods

### Outline of the economic model

A decision tree model was developed to reflect the clinical pathway of infants at high risk of severe RSV infection who either receive prophylaxis with palivizumab or no prophylaxis. Baseline risk of RSV hospitalisations and efficacy data were taken from palivizumab clinical trials. The analysis was conducted from the perspective of the UK National Health Service (NHS). Cost data were obtained from national databases and published literature. The main outcome was presented as the incremental cost per quality-adjusted life-year (QALY) gained.

The population investigated were children at high risk of RSV hospitalisation and its sequelae (chronic respiratory morbidity); i.e. preterm infants (≤ 35 wGA and aged under six months at the start of the RSV season), infants (aged under 24 months) with CLD, and infants (aged under 24 months) with haemodynamically significant CHD. These three main patient groups correspond to the licensed indications for palivizumab in Europe [7].

**Figure 1:**
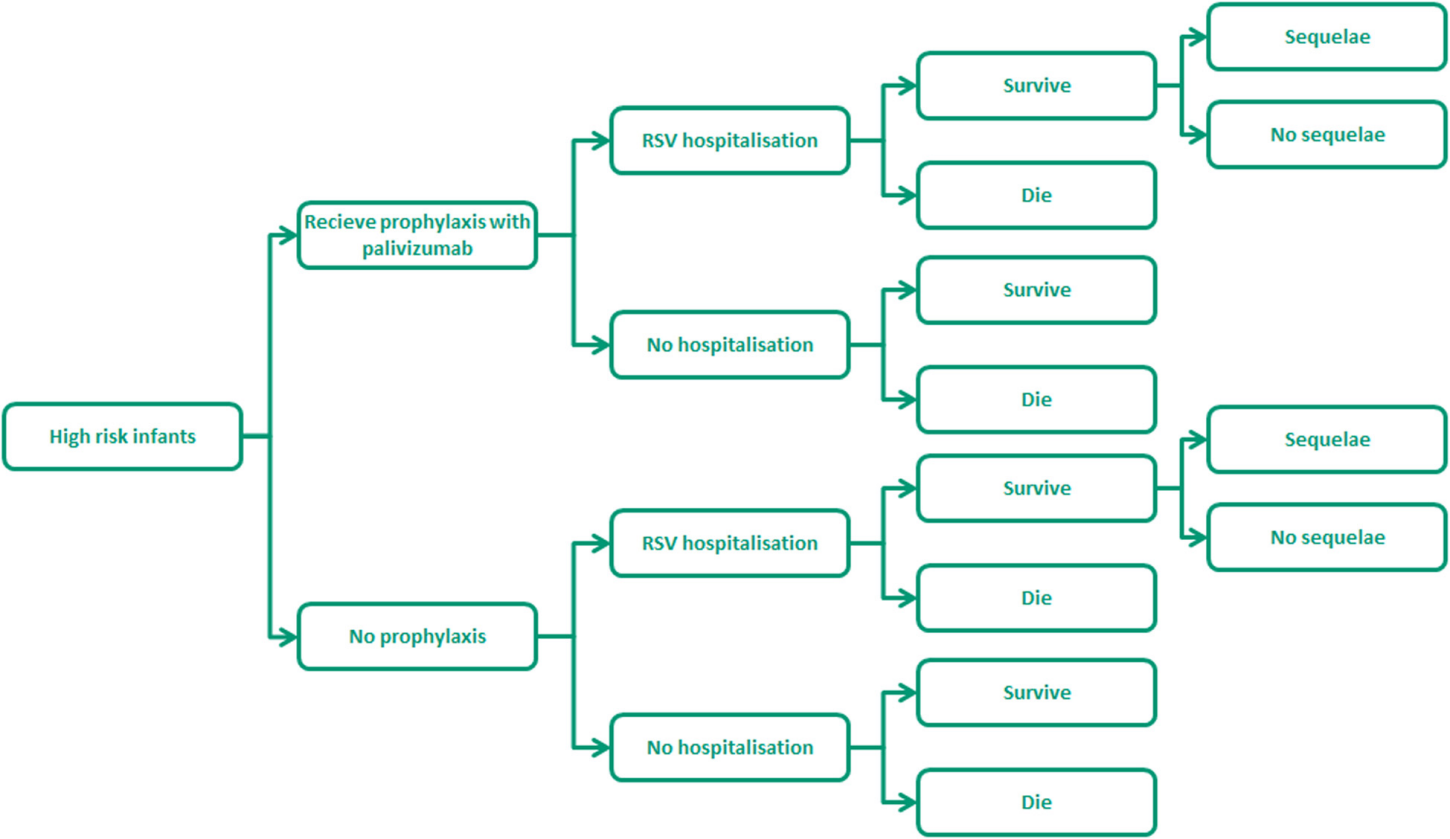
Decision tree model schematic.

The model illustrated in Figure 1 traces the pathway of infants at high risk for severe LRTI for one year, which corresponds to the RSV season (October-March in the UK) and the period of clinical follow-up. The model considers two scenarios for these high-risk infants. At the start of the season, infants either receive prophylaxis with palivizumab or no prophylaxis. The dosage of palivizumab administered is estimated based on the infant weight at the start of the season, using the clinical trial data [16,17], and an assumed increase in weight each month based on World Health Organisation (WHO) growth charts [19,20]. Both groups of infants may develop RSV infection leading to hospitalisation. The majority of these children will be managed in a paediatric ward, but some will require transfer to the Intensive Care Unit (ICU). Of the hospitalised infants, a very small proportion will die. Infants with a hospitalisation due to RSV disease will be at increased risk of developing chronic respiratory morbidity (sequelae). The base case model considers that this morbidity is likely to persist into early childhood, based on the studies by Greenough et al., and Shefali-Patel et al., which looked at the healthcare utilisation over two years in children with CLD and late preterm infants, respectively, who had RSV-proven infection requiring hospitalisation [21,22].

Both costs and outcomes were discounted at 3.5% in line with National Institute for Health and Clinical Excellence (NICE) guidelines [23]. Discount rates were varied in sensitivity analysis between 0% and 6.5%.

### Data sources used in the analysis

The model input data are summarised in Table 1.

**Table 1:** Summary of input data used in the model

**Subgroup**	**Value (lower and upper CI)**	**Reference**
**Baseline risk of hospitalisation**		
CHD infants	9.7% (7.4%, 12.0%)^†^	Feltes et al. 2003 [5]
CLD infants	12.8% (8.8%, 16.8%)^†^	IMPACT, 1998 [6]
Preterm: <29 wGA	10.00% (0.7%, 19.3%)^†^	MedImmune/Abbott, Data on File.
Preterm: 29–32 wGA	7.69% (2.86%, 12.5%)^†^	MedImmune/Abbott, Data on File.
Preterm: 33–35 wGA	7.69% (2.86%, 12.5%)^†^	MedImmune/Abbott, Data on File.
**Relative risk reduction of RSV hospitalisation with palivizumab prophylaxis**		
CHD infants	45.3% (18.1%, 63.4%)^†^	Feltes et al. 2003 [5]
CLD infants	38.5% (5.0%, 60.2%)^†^	IMPACT, 1998 [6]
Preterm: <29 wGA	80.39% (0.00%, 96.26%)^†^	MedImmune/Abbott, Data on File.
Preterm: 29–32 wGA	79.69% (35.38%, 93.62%)^†^	MedImmune/Abbott, Data on File.
Preterm: 33–35 wGA	73.16% (54.87%, 93.09%)^†^	MedImmune/Abbott, Data on File.
**Mortality rates of children hospitalised due to RSV disease**		
CHD infants	3.72% (1.19%, 6.23%)^†^	Wang et al. 2008 [14]
CLD infants	4.00% (3.00%, 5.00%)^†^	Wang et al. 2008 [14]
Preterm infants	0.43% (0.23%, 0.63%)^†^	Wang et al. 2008 [14]
**Life expectancy**		
CHD infants	76.0 years (75.0^††^, 78.9^††^)^‡^	Office for National Statistics [25]
All other subgroups	79.0 years (77.9^††^, 82.0^††^)^‡^	Office for National Statistics [25]
**Risk of RSV**-**related sequelae**		
Increase in resource use	2 years	Greenough et al. 2001 [24]
Decrease in utility	5 years	Greenough et al. 2004 [21]
**Drug and administration costs**		
Palivizumab (50 mg vial)	£306.64/pack (£6.13/mg)	MIMS [27]
Palivizumab (100 mg vial)	£563.64/pack (£5.64/mg)	MIMS [27]
Initial administration by hospital nurse	£41 (£23, £47)	Costs of Health and Social Care 2009–2010 [29]
Subsequent administration by GP practice nurse	£31 (£26, £36)	Costs of Health and Social Care 2009–2010 [29]
**Rate of hospital admissions for RSV** (% **of patients**)		
CHD children in general ward	100%	Assumption
CHD children in Intensive Care Unit (ICU)	38.14% (28.48%, 47.81%)^†^	Feltes CSR [16]
All other infants; General ward	100%	Assumption
All other infants; Intensive Care Unit (ICU)	27.45% (18.79%, 47.81%)^†^	FDA - Palivizumab Clinical Review [2]
**Length of stay of hospital admissions for RSV patients**		
CHD children in general ward	12.40 (9.30, 18.99^§^) ^‡^	FDA - Palivizumab Clinical Review [2]
CHD children in Intensive Care Unit (ICU)	15.19 (11.39, 15.50^§^)^‡^	FDA - Palivizumab Clinical Review [2]
All other infants; General ward	6.64 (4.98, 8.32^§^)^‡^	FDA - Palivizumab Clinical Review [2]
All other infants; Intensive Care Unit (ICU)	7.04 (5.28, 8.80^§^)^‡^	FDA - Palivizumab Clinical Review [2]
**Cost per day of RSV hospitalisation**		
General ward	£555^¶^ (£406*, £1,955*)	NHS National Schedule of Reference Costs [28]
Intensive Care Unit (ICU)	£2,225 ^¶^ (£311*, £1,954*)	NHS National Schedule of Reference Costs [28]
**Medical cost of sequelae** (**recurrent wheeze**/**asthma**)		
Annual per patient cost for all other subgroups	£14, 015	Greenough et al., 2004 [21]; Costs of Health and Social Care 2009–2010 [29]; NHS National Schedule of Reference Costs [28]
Annual per patient cost for 33–35 wGA	£810 (£0, £8,972)	Shefali-Petal et al., 2011 [22]
**Health state utility values** (**Utility** (**SE**); (**Lower and upper CI**)		
Non RSV-H patients	0.95 (0.25)^†^; (0.03, 1.00^‡‡^)	Greenough et al., 2004 [21]
Patients admitted with RSV-H	0.88 – Modelled as 7.37% (0.94%) reduction^†^; (5.53%, 9.21%^§^)	Greenough et al., 2004 [21]

*Abbreviations*: *CI* Confidence interval, *CHD* Congenital heart disease, *CLD* Chronic lung disease, *wGA* weeks of gestational age, *GP* General practitioner, *SE* Standard error, *RSV*-*H* Respiratory syncytial virus hospitalisation. †Beta distribution; ‡ Gamma distribution; Log-normal distribution. †† The lower and upper confidence intervals are based on the average life expectancy of males and females respectively. § Estimated assuming a confidence interval of ± 25%. * Interquartile range. ‡‡ Plausible range.

#### Clinical data

The following trials were identified to inform the clinical input data: Feltes et al. [5] considered children under 24 months of age with haemodynamically significant CHD; and the IMPACT RSV study [6], which analysed preterm infants of ≤ 35 wGA and aged less than six months at the onset of the RSV season and children under 24 months of age with or without CLD. Data from these two randomised, controlled trials (RCTs) and additional observational studies were used to inform clinical model inputs (Table 1).

#### Probability of RSV hospitalisation

Data on RSV hospitalisation risk were derived from a number of sources depending on the population subgroup as described below:

CHD children: Feltes et al. [5] reported a 9.7% risk of RSV hospitalisation among children with haemodynamically significant CHD who did not receive RSV prophylaxis (versus a 5.3% risk in the palivizumab group) and showed that the use of palivizumab for prophylaxis was associated with a relative risk reduction of 45%.

CLD children: The IMPACT RCT [6,17] found that among premature infants and children with CLD who did not receive RSV prophylaxis, the rate of RSV hospitalisation was 10.6% (versus 4.8% in the palivizumab group). Overall, monthly prophylaxis with palivizumab resulted in a 55% relative risk reduction of RSV hospitalisation. The RSV hospitalisation rate was found to be 12.8% for the subgroup of children with CLD who did not receive RSV prophylaxis (versus 7.9% in the palivizumab group), with a relative risk reduction of 39%.

Preterm < 29 wGA, 29–32 wGA and 33–35 wGA: The baseline rate of RSV hospitalisation for the preterm infant subgroups and the relative risk reduction associated with palivizumab prophylaxis were obtained from retrospective subgroup analysis of the preterm infants of the IMPACT RCT [6,17]; this subgroup analysis is based on unpublished data (MedImmune/Abbott, Data on File).

#### Probability of sequelae (recurrent wheeze asthma)

Data on the risk of RSV-related sequelae (RSV-associated respiratory morbidity) were taken from two cohort studies by Greenough et al. [21,24] and a study by Shefali-Patel [22]. These studies demonstrated that in the cohort of children with CLD [24] and 33–35 wGA babies [22] post RSV hospitalisation, there was an increase in healthcare resource use attributable to respiratory sequelae for a two-year period [24], and a decrease in quality of life experienced for five years [21]. Because the above mentioned studies consider a cohort of patients post RSV hospitalisation, the model assumes that the two-year increase in resource use and the five-year decrease in utility are applied to all infants who experience an RSV hospitalisation. No difference in the risk of sequelae was assumed between those who received prophylaxis and those who did not, as the RSV hospitalisation was a sole determinant for development of respiratory sequelae.

#### Life expectancy for infants at risk of RSV infection and probability of RSV-related mortality

The life expectancy for infants at risk of RSV infection was calculated by averaging the life expectancies for men and women, assuming an equal split, born today using data from the Office of National Statistics [25]. The mean life expectancy was estimated to be 80.0 years (standard error [SE]: 1.05). However, for the purposes of this analysis the first year of life was captured in the decision tree and so the remaining life expectancy for an infant at the age of one year was assumed to be 79.0 years. In the case of children with CHD, 95.3% were predicted to survive to age 16 years if they had survived to age of one year [26]. Therefore, the life expectancy at the age of one year was assumed to be 76.0 years for children with CHD. The mortality rates (Table 1) were as described in a recent health technology assessment by Wang et al. [14], which forms the basis for the Joint Committee on Immunisation and Vaccination (JCVI) recommendations on the usage of palivizumab [1].

#### Drug cost data

The recommended dose of palivizumab is 15 mg per kg body weight, injected intramuscularly, given once a month during anticipated periods of RSV risk in the community. The initial dose was calculated using the infant’s weight at the start of prophylaxis as reported in the RCTs [16,17]. Because no subsequent weight of infants was reported in the trial, the infant weight needs to be estimated at each month to correctly determine the subsequent prophylaxis dose. Infant weight was estimated using the UK-specific WHO growth chart data predicting infant weight based on chronological age [19,20]. Bearing in mind that the population in the model is a preterm patient population, an adjustment is required to the chronological age to account for the preterm birth. Gestational age at birth was used to derive corrected chronological age, which is then used in conjunction with the UK growth chart. The corrected chronological age refers to the age at the start of administration (as reported in two pivotal trials) adjusted to reflect the gestational age at the birth; for example, an infant who is 20 weeks old at the start of administration but was born at 30 wGA would have a corrected age of 10 weeks, assuming a typical 40-week gestation. Regression methods (ordinary least squares) were used to approximate the relationship between weight (y) and chronological age (x), using goodness of fit criteria. The resulting equation *y* = *0*.*0083x*^*3*^ - *1*.*9512x*^*2*^ + *191*.*01x* + *3869*.*4* was subsequently used to estimate the relative increase in infant weight at monthly intervals. Average age and weight at initiation of prophylaxis are shown in Table 2. In the base case analysis, the correct dose was determined based on the infant’s weight, and the number of 100-mg and/or 50-mg vials required to administer this dose were then estimated and cost determined accordingly. Drug prices were taken from the Monthly Index of Medical Specialities (MIMS) and are UK-specific [27]. The RSV season is assumed to last five months, and this also reflects the duration of administration in both palivizumab RCTs [5,6].

**Table 2:** Average age and weight at treatment initiation

**Subgroup**	**Post conception age at birth (SE)**	**Chronological age at start of administration (SE)**	Adjusted chronological age*	**Weight in g (upper and lower CI)**
CHD babies^§^	38.50 (0.10)^‡^	26.60 (0.8)	25.10	6,649 (6,257; 7,041)
CLD babies	29.00 (0.11)^‡^	23.12 (0.68)	14.13	4, 833 (4,084; 4,527)
Preterm: <29 wGA	29.00 (3.70^†^)^‡^	15.40 (0.51)	4.40	3,709 (3,503; 3,915)
Preterm: 29–32 wGA	30.50 (3.89^†^)^‡^	12.39 (0.34)	2.89	3,959 (3,804; 4,114)
Preterm: 33–35 wGA	34.00 (4.34^†^)^‡^	10.89 (0.46)	4.89	4,306 (4,084; 4,527)

*Abbreviations*: *CI* Confidence interval, *SE* Standard error, *CHD* Congenital heart disease, *CLD* Chronic lung disease, *wGA* weeks of gestational age. * Calculated assuming a normal gestation of 40 weeks; ^†^ Estimated assuming a confidence interval of ± 25%; ^‡^ Gamma distribution; ^§^ Data from Feltes et al. 2003 [4] or MedImmune Data on File.

#### Cost of RSV hospitalisation

The rate and length of hospital admissions in both the general ward and ICU were estimated using data from Feltes et al. for children with CHD [5,16], and the IMPACT study for both preterm infants and children with CLD [6,17]; however, the IMPACT study has insufficient data to determine if there is a difference in either the rate of admissions to the ICU and paediatric ward or the length of stay between the preterm subgroups or children with CLD. Therefore, for the purposes of the model they are assumed to be equal. The rate and length of hospital admissions are shown in Table 1.

The cost per day of RSV hospitalisation is shown in Table 1. Cost sources are UK-specific. Admitted patient care costs were identified from the National Schedule of Reference Costs (Trusts) 2009–2010 [28] and community care costs were identified from Costs of Health and Social Care 2009–2010 [29].

#### Medical cost of sequelae

The additional costs associated with the management of sequelae (respiratory morbidity) were applied each year to the proportion of patients suffering from these sequelae. In the base case analysis, it was assumed that all infants hospitalised with RSV disease would experience additional respiratory sequelae (respiratory morbidity) related resource use for two years compared to non-admitted infants. This is reflective of the cohort study by Greenough et al. [24] and Shefali-Patel [22], which provided the mean incremental resource use for this period for infants with CLD and 33–35 wGA, respectively. Incremental resource use for the mid preterms was extrapolated from Greenough et al. [24]. Clinical contact costs were derived from UK-specific Unit Costs of Health and Social Care 2009–2010 [29] while the unit costs of days in hospital were taken from the NHS National Schedule of Reference Costs [28]. For the purposes of modelling, a single cost input was used for respiratory sequelae-associated resources. For the 33–35 wGA subgroup, this cost was taken directly from Shefali-Patel et al. [22] at £1,342 over two years; this figure was inflated to 2010 costs and halved to give an annual figure of £810. For all other subgroups, a cost of £14,015 per patient per year was used, calculated from the product of the units of resource use and the associated unit costs (less the cost of the initial RSV hospitalisation, omitted to avoid double counting) reported by Greenough et al. [24]. This approach and the resultant costs reflect those presented by Wang et al. [13,14], however the alternative data source for the 33–35 wGA subgroup results in a significantly different sequelae-associated determination of resources compared to the other subgroups. It is uncertain if this difference is due to an actual difference in the rate of sequelae between subgroups or due to the methodologies used to capture and report the resource use between the studies. Using the lower figure for the 33–35 wGA subgroup is a conservative assumption. A single cost input was used to avoid overestimating the impact of individual cost components associated with respiratory sequelae in univariate (one-way) sensitivity analysis.

#### Quality of life

The health-related quality of life (HRQoL) values used in the model are shown in Table 1 and assume a disutility associated with RSV hospitalisation and respiratory sequelae five years down the line. Utility data were obtained from the study by Greenough et al. [21].

This retrospective study assessed HRQoL in infants 5 years of age who had previously been admitted to a neonatal admissions service. This study administered the multi-attribute Health Utilities Index Mark 2 (HUI2) and HUI3 instruments to assess health status and HRQoL. These instruments were sent to parents who were asked to make an assessment of the child’s HRQoL over the previous 4 weeks. Participants comprised 190 infants with CLD and a median wGA of 27 weeks (range 22–33 weeks), retrospectively identified as having stayed in a neonatal admissions service. Of these 190 patients, 33 had proven RSV infection. The HUI2 was originally developed for paediatric application and clinical evaluation studies, whereas HUI3 was developed for use in adults and population surveys. As such, the utilities obtained using the HUI2 measure has been used in the model over the HUI3. For the purposes of the model, these same utilities have been applied for the immediate RSV hospitalisation and those associated with long-term sequelae. Since HRQoL was assessed at five years of age in the Greenough et al. study, the model conservatively assumes that the decrement in HRQoL attributable to respiratory sequelae is also only applied for a five-year period.

### Model validation

#### One-way sensitivity analysis

In the one-way (univariate) sensitivity analysis, all model parameters were individually varied between their minimum and maximum values, based on the 95% confidence interval for all parameters, and the ICER recorded. These results are presented in the form of a tornado diagram; however, for simplicity only the 10 parameters that have the greatest impact on ICER variability are presented.

#### Probabilistic sensitivity analysis (PSA)

PSA was performed using Monte Carlo simulation techniques that allow all parameters to be varied simultaneously within a plausible range. The probability of RSV hospitalisation, prophylaxis efficacy, and mortality were given beta distributions. Beta distributions were also applied to the baseline utility scores for RSV and non-RSV hospitalisation. The estimation of the distribution ranges was based on published data or, where unavailable, the confidence interval was assumed to equal 25% of the deterministic value and the standard errors were calculated.

All costs were assigned a gamma distribution, as this takes into account the likely skew and variability with these parameters. The lowest and highest prices per day were used as estimates for the credible range and used to estimate a standard error. For all other costs, the confidence interval was assumed to equal 25% of the deterministic value and the standard errors were calculated.

Other parameters include the number of prophylaxis doses, which varied between three and six doses, the duration to which to apply the incremental costs associated with respiratory sequelae, varied between zero and five years, and utility differences associated with respiratory sequelae, varied between three and six years. A gamma distribution was assigned to these parameters.

## Results

### Base case

Table 3 presents the base case results for the cost-effectiveness analysis and shows the total costs and QALYs associated with prophylaxis with palivizumab and no prophylaxis based on hypothetical cohorts of 100 infants within each of the subgroups. Prophylaxis against severe RSV infection results in ICERs of £33,216, £19,168, £3,845, £30,205 and £99,056 per QALY for high-risk infants with CHD, infants with CLD, premature infants <29 wGA, premature infants 29–32 wGA, and premature infants 33–35 wGA, respectively, compared with no prophylaxis.

**Table 3:** Summary of results per hundred infants from the base case model

	**Palivizumab**		**No prophylaxis**		
	**Costs**	**QALYs**	**Costs**	**QALYs**	**ICER**
CHD infants	£636,108	2,597	£449,120	2,591	£33,216
CLD infants	£569,491	2,613	£440,816	2,606	£19,168
Preterm infants					
<29 wGA	£367,776	2,622.94	£354,226	2,619.42	£3,845
29-32 wGA	£353,668	2,623.12	£272,481	2,620.43	£30,205
33-35 wGA	£318,079	2,622.90	£73,621	2,620.43	£99,056

*Abbreviations*: *CHD* Congenital heart disease, *CLD* Chronic lung disease, *wGA* weeks of gestational age, *QALY* Quality-adjusted life-year, *ICER* Incremental cost-effectiveness ratio.

### One-way sensitivity analysis

**Figure 2:**
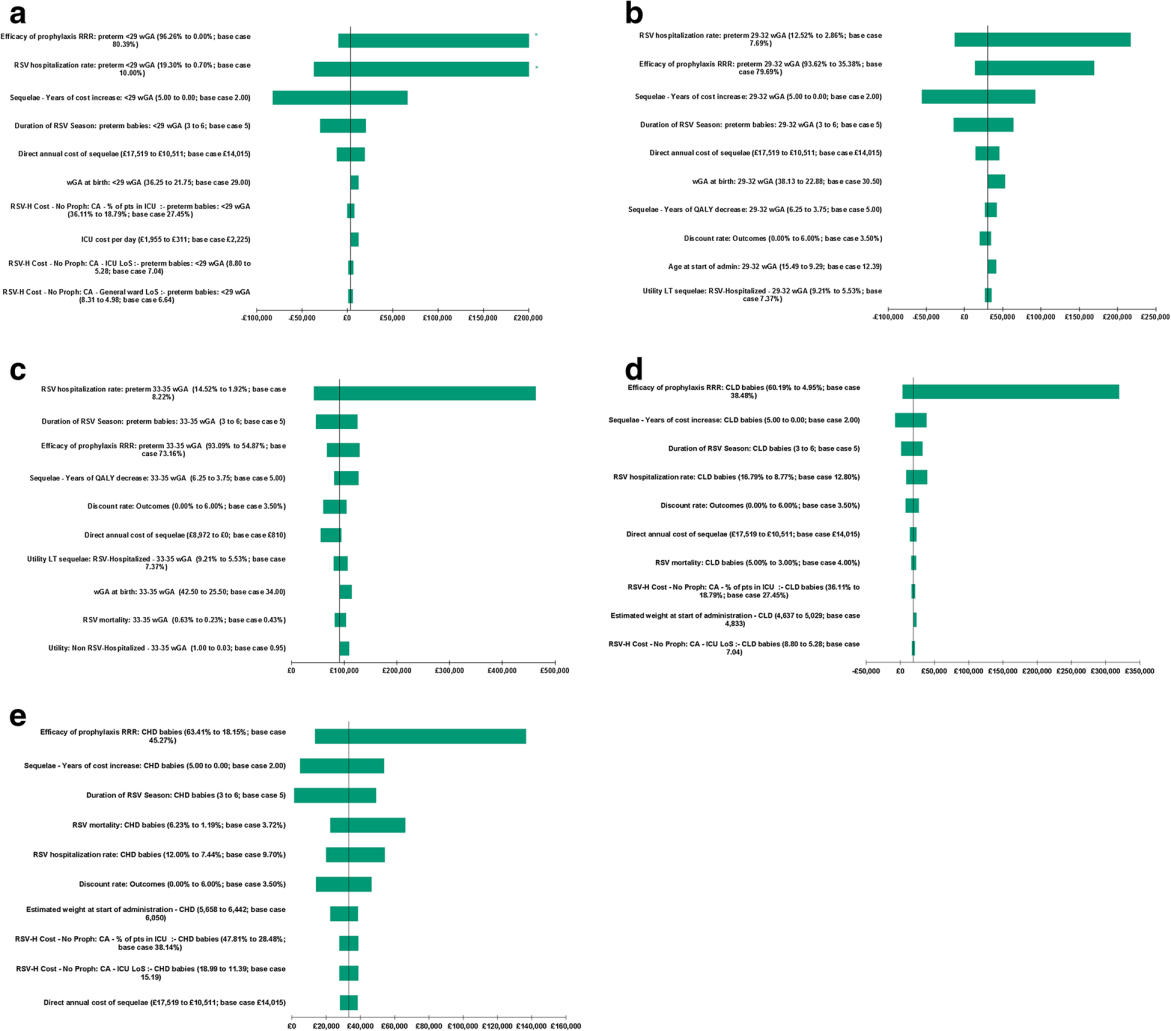
**Univariate sensitivity analysis of top 10 parameters by subgroup. a**: preterm infants < 29 weeks. **b**: preterm infants 29–32 wGA. **c**: preterm infants 33–35 wGA. **d**: CLD infants. **e**: CHD infants.

Univariate sensitivity analysis demonstrates how the ICER is affected by varying parameters through a range of extremes, as illustrated in Figure 2. Depending on the parameter altered, the ICER ranges from greater than the accepted willingness to pay (WTP) threshold of £30,000 per QALY to dominant (clinically superior and cost saving). The influence of two parameter types was common across all subgroups: those affecting the number of RSV hospitalisations (such as the underlying risk of RSV hospitalisation, the efficacy of palivizumab prophylaxis, and the duration of the RSV season) and those affecting costs (such as the weight and/or age of infants at the start of administration). Other significant drivers across the subgroups include, to a varying degree, the cost of managing respiratory sequelae, the number of years to apply lower utility for or increased healthcare consumption associated with sequelae, and the discount rate associated with outcomes.

### PSA

**Figure 3:**
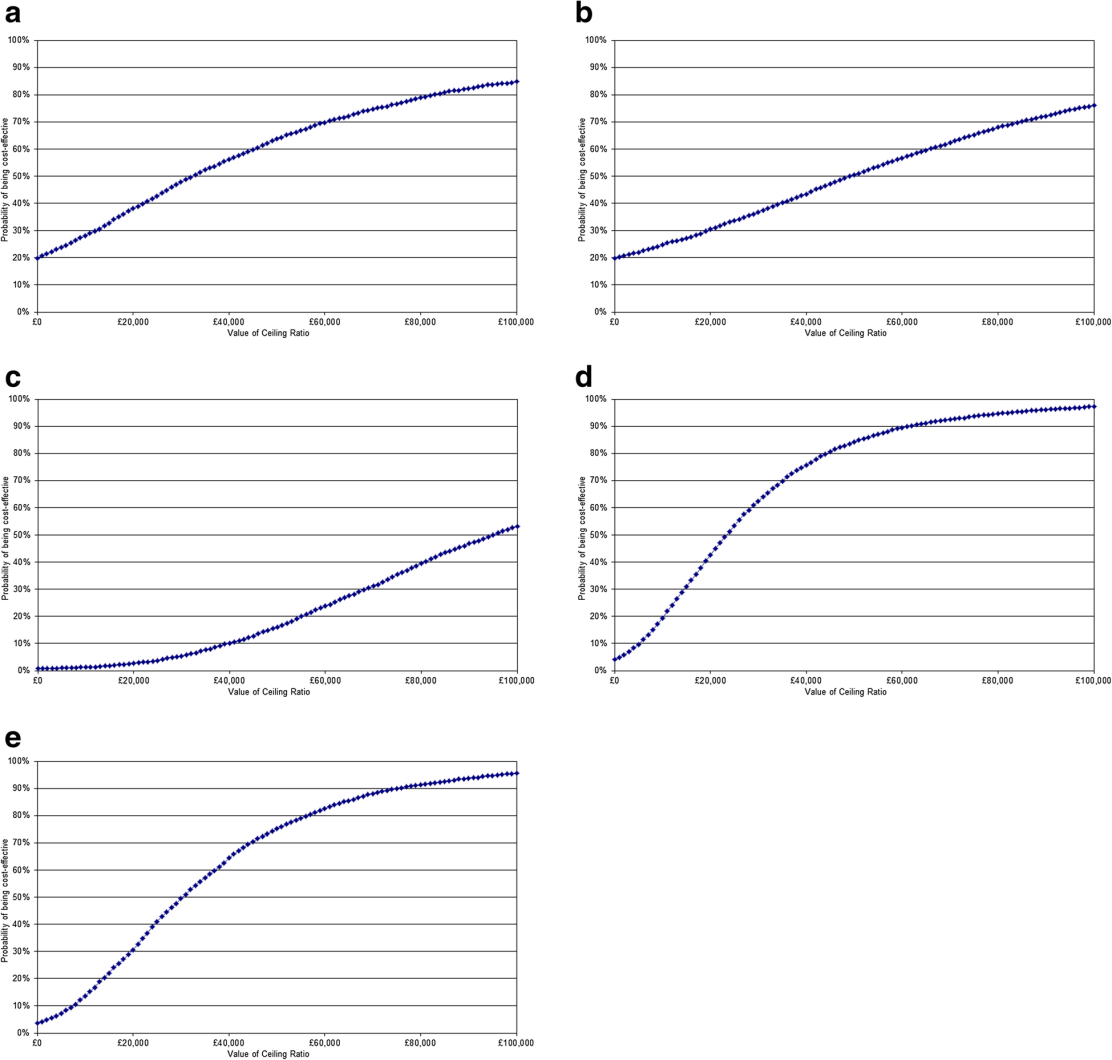
**Cost**-**effectiveness acceptability curves by subgroup. a**: preterm infants <29 wGA. **b**: preterm infant’s 29–32 wGA. **c**: preterm infant’s 33–35 wGA. **d**: CLD infants. **e**: CHD infants.

Table 4 shows the probability that the intervention is cost-effective, based on the results of 5,000 Monte Carlo simulations at a WTP threshold of £20,000 and £30,000 per QALY. While the cost acceptability of palivizumab may be challenged at the £20,000 threshold, since all subgroups have a less than 50% probability of being cost-effective, the results are more favourable at the £30,000 threshold. The associated cost-effectiveness acceptability curves (CEACs) are shown in Figure 3. The CEAC curve plots the proportion of simulations that will be cost-effective as the WTP threshold is varied along a continuum.

**Table 4:** **Probability that palivizumab is cost**-**effective for different WTP thresholds**

**Sub-group**	**WTP threshold £20,000**	**WTP threshold £30,000**
CHD	36.76%	51.52%
CLD	45.04%	60.06%
<29 wGA	45.02%	51.22%
29-32 wGA	30.44%	36.78%
33-35 wGA	2.8%	5.4%

*Abbreviations*: *CHD* Congenital heart disease, *CLD* Chronic lung disease, *wGA* weeks of gestational age, *WTP* Willingness to pay.

### Risk factors in the preterm infant’s 33–35 wGA subgroup

The model estimates an ICER of £99,056 per QALY for preterm infants in the 33–35 wGA subgroup. While this is higher than the level conventionally accepted by NICE in the UK, it is plausible that a proportion of infants in this subgroup (i.e. those most “at risk”) would benefit from palivizumab prophylaxis. Therefore a further threshold analysis was performed to investigate the impact of considering additional RSV-hospitalisation risk factors. Various clinical and environmental factors, such as the number of siblings, male gender and parental smoking [30-33], are known to increase the risk of RSV-hospitalisation. However, individual patients may have one or more of these factors and so considering them in isolation would be of limited value. Furthermore, there is considerable variance between data sources regarding the prevalence of environmental risk factors such as parental smoking, day care attendance and breast feeding. Additionally, studies of interest vary in quality, sample size, design and population investigated. Therefore, it was deemed that the best approach in this threshold analysis was to simply consider the impact of incrementally increasing the overall risk of RSV-hospitalisation and its effect on the ICER.

**Figure 4:**
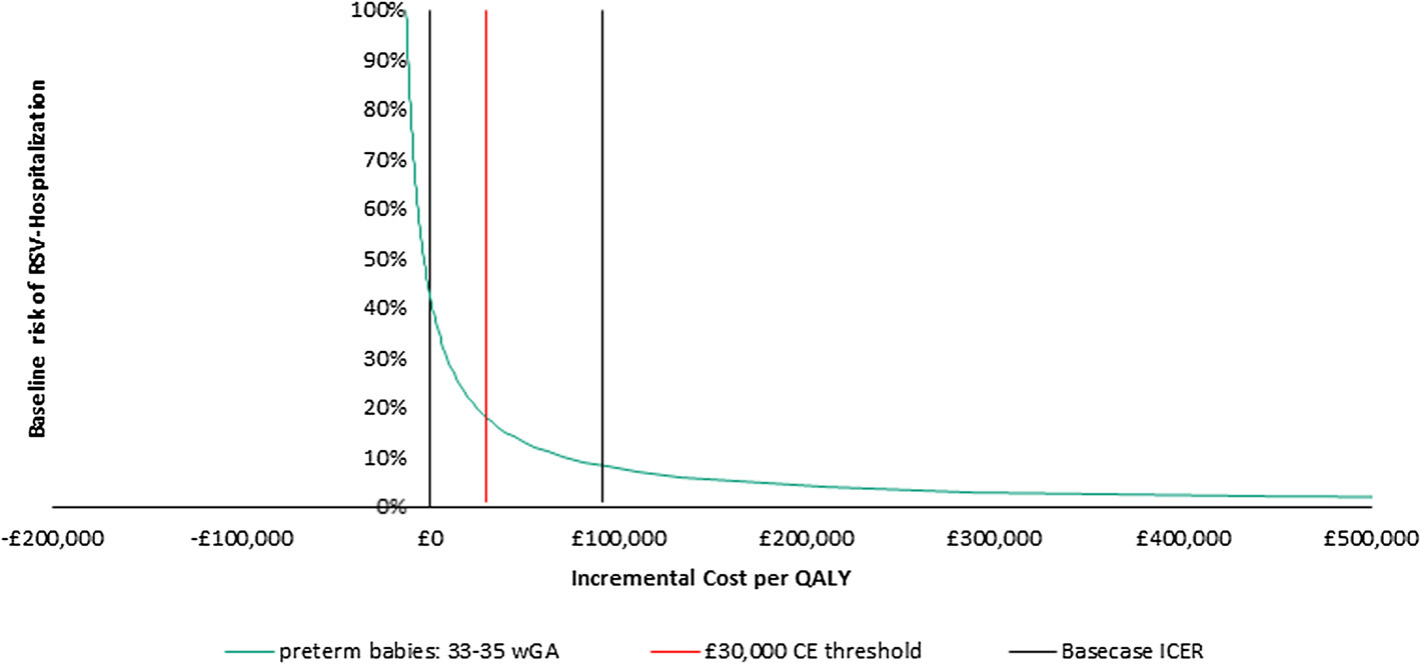
Effect of risk of RSV hospitalisation on cost-effectiveness for preterm infants 33–35 wGA.

Figure 4 shows the effect of baseline risk of RSV hospitalisation on cost-effectiveness for preterm infants 33–35 wGA. The curve shows how varying the risk affects the acceptability of the ICER. For instance, at a WTP of £30,000/QALY, palivizumab prophylaxis is cost-effective for an infant with an adjusted baseline risk of RSV hospitalisation of at least 17.94%. Similarly, prophylaxis is cost-effective at the £20,000/QALY threshold if the risk of RSV hospitalisation is greater than 22.23%.

## Discussion

The results of this economic analysis have shown that prophylaxis with palivizumab represents good use of NHS resources for children at high risk of RSV-related hospitalisation and its sequelae in the UK. Prophylaxis with palivizumab is cost-effective in infants with CLD or <29 wGA at cost/QALY of £19,168 and £3,845 respectively. Cost-effectiveness in 29–32 wGA preterm and CHD infants was demonstrated at £30,205 and £33,216 without additional risk factors being considered. The slightly higher mean starting weight and age for CHD infants resulting from the broader treatment eligibility of ≤ 24 months compared to ≤ 6 months for preterm infants led to higher prophylaxis costs, which likely explains the ICER being greater than in other subgroups. Cost/QALY of £99,056 was observed for 33–35 wGA preterm infants; however, sensitivity analysis demonstrated that prophylaxis becomes cost-effective at the £30,000 threshold in those at the highest risk of RSV hospitalisation. The analysis shows that palivizumab would represent a cost-effective use of NHS resources for a vulnerable and important patient population. Similar approaches to vaccination are utilised in the Bacillus Calmette-Guérin (BCG) national immunisation programme in the UK targeting infants most at risk of exposure to tuberculosis [34].

Sensitivity analyses illustrated that subgroups are susceptible to different parameter variations. However, common drivers of cost-effectiveness included those affecting the number of RSV hospitalisations and those affecting costs. Unsurprisingly, the discount rate associated with outcomes was identified as a significant factor for many scenarios, due to the upfront cost of prophylaxis followed by a prolonged health gain in the future. Historically, mortality associated with RSV hospitalisation was identified as a key parameter of uncertainty. While this is the case for some subgroups in this analysis, it is not the case throughout given the relatively low base case mortality rates assumed, especially for the preterm infant groups.

### Strengths and limitations

The main limitations of the study arose from the uncertainty surrounding the epidemiology of RSV-associated hospitalisation, mortality, and sequelae. RSV is not routinely tested for across hospitals and, therefore, it is difficult to gauge the true hospital burden and mortality rate associated with the disease. Rates of RSV-associated respiratory sequelae are also very difficult to ascertain. Increasing retrospective studies suggest an association of severe RSV infection with consequences such as asthma [9,35]; however, it is unclear whether this is a direct cause nor whether RSV disease prevention will reduce such consequences. Overall, further large, prospective, observational studies would be beneficial for providing estimates of RSV-associated rates of hospitalisation, mortality and sequelae.

A key strength of this analysis is that it utilises updated efficacy data derived from the IMPACT pivotal trial and as much UK-specific data as possible. Furthermore, the analysis is based on a set of conservative estimates, which may underestimate the cost-effectiveness of palivizumab. First, all infants are assumed to have received five doses of palivizumab; however, infants born after the onset of the RSV season would require fewer than five doses. In addition, only costs borne by the health care sector, such as medication costs and general practitioner and hospital visits, were included in the analysis; the inclusion of societal cost, such as costs due to out-of-pocket expenses or lost productivity while the parent cares for the child, or any RSV cases that required ambulatory care may contribute to lower ICER results.

### Previous evaluations

A recent systematic review has shown that ICERs vary greatly from study to study, making it difficult for decision makers to decide whether prophylaxis with palivizumab is cost-effective [14]. Differences in populations, interventions, perspectives, and time horizons have all contributed to the discrepancies between studies. Wang et al. [13,14] and Nuijten et al. [12] have reported the cost-effectiveness of palivizumab from a UK perspective. Nuijten et al. suggested that palivizumab may be cost-effective in preterm infants ≤ 35 wGA, infants with bronchopulmonary dysplasia, and infants with CHD [12]. On the other hand, Wang et al. reported that prophylaxis with palivizumab does not represent good value when used unselectively in preterm infants without CLD or infants with CLD or CHD; however, it may be cost-effective (based on a threshold of £30,000/QALY) for infants with CLD or CHD when they have two or more additional risk factors [14]. Several previous economic evaluations have attempted to model the impact of risk factors for RSV hospitalisation on cost-effectiveness [13,14]. Studies suggest an association between hospitalisation with RSV and clinical/environmental risk factors such as gestational age, age at the commencement of the RSV season, birth weight, gender, number of smokers in the household, and having siblings [14,32,33]. Some of these economic evaluations have used methods for synthesising the risk factors into a single model, which may introduce bias and complexity as they combine multiple risk factors without consideration of possible interactions between risk factors. In our economic analysis, a threshold analysis has been developed that considers the overall risk of RSV hospitalisation rather than attempting to identify the impact of specific individual clinical and environmental risks factors themselves. This approach allows the demonstration of a relationship between level of the baseline risk of RSV hospitalisation and cost-effectiveness of palivizumab irrespective of the specific combinations of risk factors. By relying on the overall risk of RSV hospitalisation, clinicians or decision makers are empowered to use whichever risk factor assessment they deem appropriate to estimate the baseline risk of RSV hospitalisation.

## Conclusion

Using updated efficacy data derived from the pivotal IMPACT trial, most recent healthcare costs, and making conservative assumptions, the current analysis demonstrates the cost-effectiveness of palivizumab versus no prophylaxis in infants at high risk of hospitalisation with RSV in the UK. Therefore, prophylactic palivizumab represents an economically viable use of NHS resources for infants (aged under 24 months) with CHD, infants (aged under 24 months) with CLD and preterm infants born at 32 wGA or below and preterm infants born 33–35 wGA when additional risk factors are considered.

## Competing interests

IF, KG and KB are employees of AbbVie (previously Abbott). AB is an employee of Abacus, which received funding from AbbVie for this study.

## Authors’ contributions

AB was responsible for design of study, acquisition of data, analysis and interpretation of the results, drafting and critically revising the manuscript, received funding, IF was responsible for design of study, acquisition of data, interpretation of the results, drafting and critically revising the manuscript, KG is an was responsible for conception and design of study, interpretation of the results, drafting and critically revising the manuscript, KB was responsible for concept and design of study, interpretation of the results, drafting and critically revising the manuscript. All authors read and approved the final manuscript.
